# FOXN3 inhibits cell proliferation and invasion via modulating the AKT/MDM2/p53 axis in human glioma

**DOI:** 10.18632/aging.203499

**Published:** 2021-09-12

**Authors:** Chaojia Wang, Hanjun Tu, Ling Yang, Chunming Ma, Juntao Hu, Jie Luo, Hui Wang

**Affiliations:** 1 Department of Neurosurgery, Taihe Affiliated Hospital of Hubei University of Medicine, Shiyan 442000, China; 2 First School of Clinical Medicine, Hubei University of Medicine, Shiyan 442000, China; 3 Department of Pediatrics, Taihe Affiliated Hospital of Hubei University of Medicine, Shiyan 442000, China; 4 Department of Rehabilitation, Taihe Affiliated Hospital of Hubei University of Medicine, Shiyan 442000, China

**Keywords:** FOXN3, cell proliferation, cell migration, glioma, AKT/MDM2/p53 pathway

## Abstract

This study aimed to evaluate the biological role of forkhead box N3 (FOXN3) in human glioma and clarify the possible molecular mechanisms. FOXN3 expression patterns in clinical tissue specimens were characterized via qPCR and Western blotting. Kaplan-Meier survival curve was applied to assess the correlation between FOXN3 expression and overall survival. Effects of FOXN3 over-expression and depletion on glioma cell proliferation, apoptosis, migration and invasion were assessed by CCK8, colony formation assay, flow cytometry, scratch wound healing assay and Transwell invasion assay, respectively. Moreover, the involvement of AKT/murine double minute 2 (MDM2)/p53 pathway was evaluated. Additionally, tumor transplantation model assay was performed to determine the effects of FOXN3 over-expression on glioma cell growth *in vivo*. Results showed that FOXN3 was significantly down-regulated in glioma tissues compared with normal tissues. Patients with lower FOXN3 expression exhibited a shorter overall survival time. Gain- and loss-of-function analyses demonstrated that FOXN3 over-expression significantly suppressed proliferation, survival and motility of glioma cells, whereas FOXN3 knockdown remarkably promoted glioma cell proliferation, survival and motility. Furthermore, FOXN3 over-expression inhibited the activation of AKT/MDM2/p53 signaling pathway in glioma cells, while FOXN3 depletion facilitated its activation. Additionally, tumor xenograft assays revealed that FOXN3 over-expression retarded glioma cell growth *in vivo*. Collectively, these findings indicate that FOXN3 inhibits cell growth and invasion through inactivating the AKT/MDM2/p53 signaling pathway and that FOXN3-AKT/MDM2/p53 axis may represent a novel therapeutic target for glioma patients.

## INTRODUCTION

Human glioma has emerged as one of the most common intracranial malignancies in recent decades, accounting for roughly 45% of all brain tumors [1–3]. It is estimated that approximately 15,000 cancer-related deaths were attributed to glioma annually in United States [4–6]. In spite of the fact that great advances regarding the standardized therapies have been made for glioma patients, the long-term prognosis of malignant glioma patients is still pretty frustrating [7–9]. Thus, there is an urgent need to better understand the exact mechanisms underlying glioma development, which could facilitate to develop effective treatment strategies and improve the prognosis for glioma patients.

It is well documented that Forkhead box N3 (FOXN3) belongs to the FOX family of transcriptional factors, which are implicated in a great variety of biological activities, including cell survival, cell proliferation and cell migration as well as cell invasion [10–12]. Recent studies have uncovered that FOXN3 functions as a key player in multiple kinds of malignant tumors, such as papillary thyroid cancer [13], tongue squamous cell carcinoma [14], melanoma [15] and breast cancer [16]. Nevertheless, potential biological functions of FOXN3 in human glioma remain largely unclear.

The current study aimed to explore potential biological functions of FOXN3 in human glioma and clarify possible molecular mechanism. Herein, it was found that FOXN3 was markedly under-expressed in glioma tissue samples and glioma cell lines. Furthermore, gain of function and loss of function researches were applied to explore effects of FOXN3 on the proliferation, colony formation and motility capabilities of glioma cells. In addition, the involvement of AKT/murine double minute 2 (MDM2)/p53 axis was also assessed. Taken together, the present study offers novel insights into elucidating biological functions of FOXN3 in glioma.

## MATERIALS AND METHODS

### Clinical sample collection

Sixty-three glioma patients who received surgical operation were recruited in Taihe Affiliated Hospital of Hubei University of Medicine (Shiyan, China) from February 2013 to December 2018. Tumor tissue samples were obtained from patients with glioma. Normal non-tumorous brain tissue samples were collected from sixty-three volunteers who suffered from traffic accidents. Written informed consents were gathered from all the participants prior to their enrollment in this study. The fresh clinical tissue specimens were immediately placed in an ultra-low temperature freezer (–80°C) for subsequent studies. The present study gained approval from Ethics Committee of Taihe Affiliated Hospital of Hubei University of Medicine (IRB approval No. 2012090058).

### Cell culture and cell transfection

Four glioma cell lines (LN229, SF126 and U87MG as well as U251MG) and HEB cells (normal human gliocytes) were obtained from Shanghai Cell Institute of Chinese Academy of Sciences. Cells were incubated in RPMI 1640 medium (Gibco, Grand Island, NY, USA) containing 10% fetal bovine serum (FBS). Cells were cultured in a 5% carbon dioxide humidified atmosphere at 37°C.

Lipofectamine 3000 (Invitrogen, Carlsbad, CA, USA) was applied to carry out cell transfection assay according to manufacturer’s instructions. The pcDNA3.1 and pLKO.1 plasmids were used for over-expression and knockdown studies, respectively. FOXN3 expression vectors and short hairpin RNAs (shRNAs) specially targeting FOXN3 were constructed in Shanghai GenePharma Co. Ltd (Shanghai, China). Sequence information concerning sh-NC and shRNA#1 as well as shRNA#2 was listed as followed: sh-NC, 5′-CGCGTCCCGTGGATATTGTTCAAGAGAAATATCCACTTGGAATAGCAA-3′; shRNA#1, 5′-CACCGCC TGACATCCGATTAGAAGACGAATCTTCTAATCGGATGTCAGG-3′; shRNA#2, 5′-CACCGCCTCATAT TTATGGCCATCGCGAACGATGGCCATAAATATGAGGC-3′. Transfection efficiency was evaluated via Western blotting analysis at 48 h post-transfection.

### Real-time qPCR analysis

TRIzol was used to exact total RNAs from clinical tissue samples using in line with manufacturer’s protocols (Takara, Dalian, China). TaqMan High-Capacity cDNA Reverse Transcription Kit was applied to synthesize complementary DNA strand (Applied Biosystems, Carlsbad, CA, USA). Afterwards, qPCR analysis was conducted using the SYBR Green PCR Kit (Applied Biosystems) on the ABI 7500 Fast Real-time PCR System. Relative expression level of FOXN3 mRNA was calculated via the 2^–ΔΔCt^ method. Herein, β-actin acted as an endogenous reference gene. Sequence information about specific primers was provided as followed: FOXN3, upstream 5′-CCGGACAGTGGGA ACTAACT-′3 and downstream 5′-GCTGTGGATGGC TTTTAGGG-′3; β-actin, upstream 5′-GTGGGGCGCC CCAGGCACCA-′3 and downstream 5′-CTCCTTAAT GTCACGCACGATTTC-′3. Relative FOXN3 mRNA expression was determined via triplicate experiments.

### MTT assays

MTT Cell Proliferation Assay Kit (Invitrogen) was applied to evaluate cell proliferation ability. MTT assays were performed based on manufacturer’s protocols. Briefly, 20 μl of MTT solution was placed into the 96-well plates and was then cultured for 4 hours at 37°C. Afterwards, 150 μl of DMSO solutions was added into the 96-well plates after discarding the supernatants. Finally, a Spectramax M5 spectrophotometer was used to detect the absorbance at 490 nm. All experiments were conducted in triplicates.

### Colony formation ability analysis

Clonogenic capability of tumor cell was assessed through colony formation assays. Briefly, tumor cells were placed into the six-well plates at the concentration of 1 × 10^3^ cells per well and were incubated for two weeks. Fifteen minutes’ incubation with 10% formaldehyde was used to fix the forming colonies, followed by 0.1% crystal violet staining. In the end, the stained colonies were observed and were counted via an inverted microscope. Clonogenic ability was evaluated by triplicate experiments.

### Cell apoptosis detection

Tumor cell apoptosis was analyzed using FITC-Annexin V/PI Apoptosis Detection Kit according to manufacturer’s guidelines. Briefly, collected cells were resuspended in the binding buffer (0.5 ml) after being washed twice by cold PBS. The cells were maintained in the dark for 15 minutes at room temperature followed by addition of 5 Annexin V-FITC solution (5 μl) and PI solution (5 μl). Finally, flow cytometry was applied to detect the apoptotic cells. All the experiments were conducted in triplicates.

### Scratch wound healing assays

The scratch wound healing assays were used to determine the Migration ability of tumor cells were determined by wound healing assays. Briefly, about 1000 cells per well were seeded into the 6-well plates. The cells were scratched using a sterile 200 μl pipette tip when reaching a confluence of 100% and were incubated in the culture medium for 24 hours. Subsequently, the wound closure was observed under a microscope. Migration abilities of tumor cells were assessed via triplicate experiments.

### Transwell invasion assay

Invasive ability of tumor cells was examined by Transwell invasion method. In brief, about 2 × 10^4^ cells were placed into the upper chamber which contained fetal bovine serum-free culture medium. Matrigel (BD Bioscience, San Jose, CA, USA) was applied to coat the upper chamber which was filled with DMEM culture with 10% fetal bovine serum. Cells were then incubated for twenty-four hours at 37°C. Subsequently, the invading cells were fixed using paraformaldehyde (4%) and were visualized using crystal violet. Finally, the stained cells were observed and were photographed. Invasion capabilities of cells were analyzed through triplicate experiments.

### Tumor xenograft assay in nude mice

Animal experiment was carried out in Taihe Affiliated Hospital of Hubei University of Medicine. Animal experiment gained approval from the Animal Care and Use Committee of Taihe Affiliated Hospital of Hubei University of Medicine (IACUC approval No. 2019120746). Ten BALB/c nude mice (male) were obtained from Beijing HFK Bioscience Co. Ltd (Beijing, China) and were classified into 2 groups (*n* = 5). Tumor xenograft models were constructed via inoculation of empty vector or FOXN3 expression vector-treated U87MG cells (1 × 10^6^ cells per mouse) into the flanks of nude mice. Volumes of formed tumors was determined every seven days based on the calculation formula that tumor seize (mm^3^) = 0.5 × width^2^ (mm^2^) × length (mm). All the mice were anesthetized via isoflurane inhalation and were sacrificed via cervical dislocation at day 28 post-transplantation. Then the tumors were peeled and weighed.

### Western blotting analysis

Total proteins were extracted from tissue samples and cell samples using the lysis buffer containing protease inhibitor. The concentrations of extracted protein samples were measured via BCA Protein Assay Kit (Takara, Dalian, China), followed by 10% SDS-PAGE separation. Then protein samples were transferred onto PVDF membrane, followed by one hour’s 5% non-fat milk incubation. Primary antibodies were then incubated overnight at 4°C. The primary antibodies (Abcam, Cambridge, MA, USA) were used at the following dilutions: anti-β-actin (1:2000), anti-FOXN3 (1:1000), anti-PCNA (1:1000), anti-CCND1 (1:1000), anti-cleaved caspase 3 (1:1000), anti-Bcl2 (1:1000), anti-MMP9 (1:1000), anti-Vimentin (1:1000), anti-AKT (1:1000), anti-p-AKT (1:1000), anti-MDM2 (1:1000) and anti-p53 (1:1000). Afterwards, the secondary antibody was incubated for one hour. ECL Western Blotting Detection Kit was purchased from Amersham Bioscience (Piscataway, NJ, USA) and was used to visualize protein bands.

### Immunohistochemical staining

Immunohistochemical staining was carried out as previously described. Briefly, 4 μm-thick paraffin-embedded tissue sections were subjected to deparaffinization and rehydration, followed by antigen retrieval. Subsequently, endogenous peroxide activity was quenched via incubating tissue slices with 3% H_2_O_2_. Non-specific binding was blocked through incubation of bovine serum albumin. Primary antibodies (anti-Ki67, anti-cleaved caspase 3 and anti-Vimentin) were then incubated with tissue sections at 4°C overnight, followed by one hour’s incubation with horseradish peroxide (HRP)-labeled secondary antibody. The slides were visualized via diaminobenzidine (DAB) staining. The positive staining in the tissue slices was were photographed and analyzed.

### Data analysis

SPSS v20.0 was used to conduct data analysis. Results were presented as mean ± standard deviation. The student’s *t-*test was used to analyze the differences between two groups. One-way ANOVA and Dunnett’s post-hoc comparison were performed to evaluate differences among three or more groups. Kaplan-Meier survival curve was adopted to determine the link between FOXN3 expression and overall survival time of glioma patients. A *P*-value of less than 0.05 indicated significantly different.

## RESULTS

### Decreased FOXN3 expression is observed in glioma tissues

**Figure 1:**
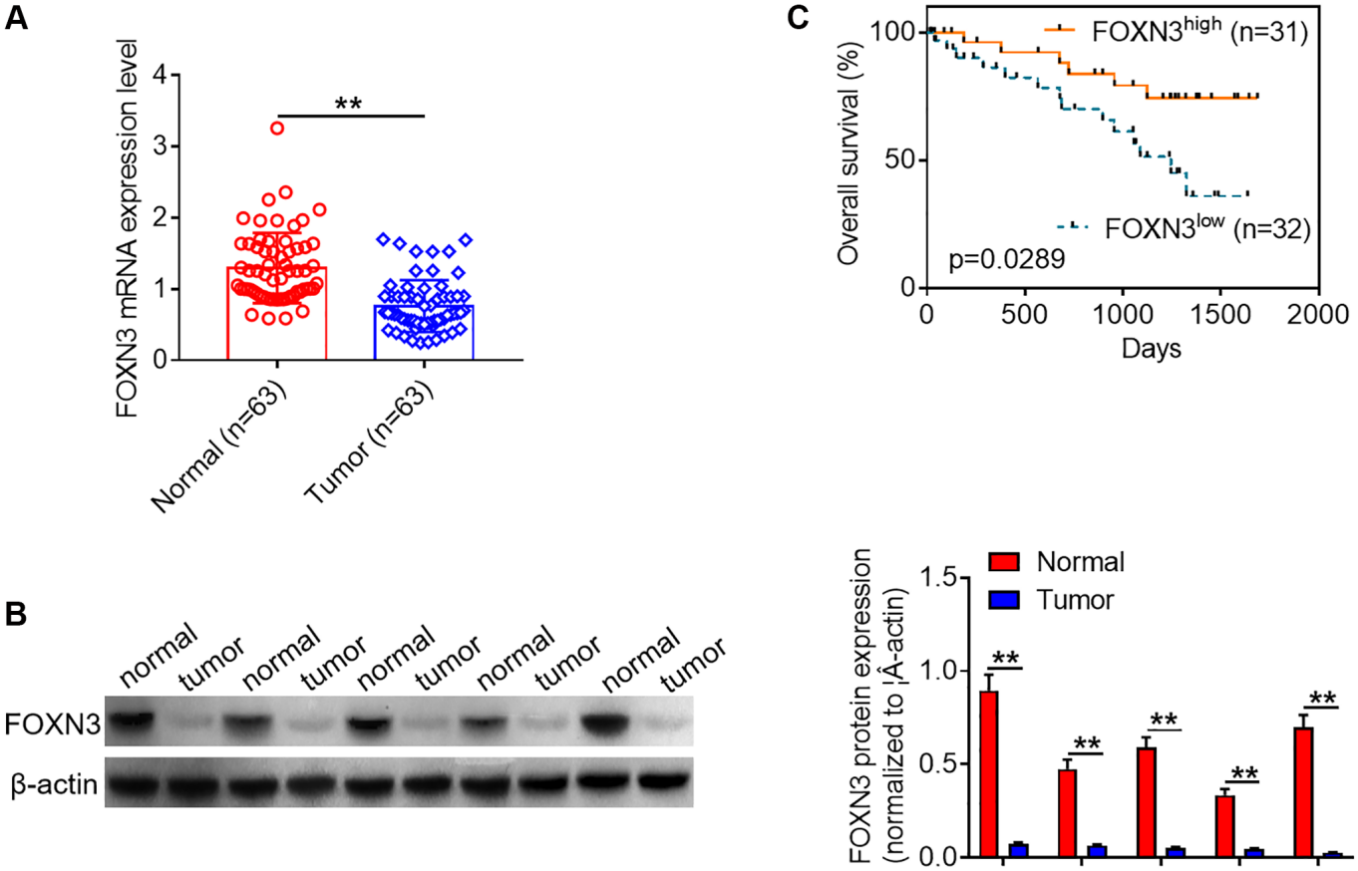
**Decreased FOXN3 expression is observed in glioma tissues.** (**A**) FOXN3 mRNA expression levels in clinical tissue samples were determined via qPCR analysis. (**B**) FOXN3 protein expression levels in clinical tissue samples were examined using Western blotting. (**C**) Glioma patients were divided into high FOXN3 expression group and low FOXN3 expression group based on the median of its expression levels in cancerous tissues. Kaplan-Meier survival curve was used to analyze the correlation between FOXN3 mRNA expression level and overall survival time. ^**^P < 0.01. Abbreviation: FOXN3: forkhead box N3.

In order to explore the role of FOXN3 in human glioma, qPCR analysis was performed to characterize its expression patterns in glioma tissues and healthy brain tissues. As shown in Figure 1A, FOXN3 mRNA expression were significantly decreased in the glioma tissues in comparison with normal brain tissues (*P* < 0.01). In consistence with the findings about FOXN3 mRNA expression, Western blotting analysis showed that FOXN3 protein was notably under-expressed in glioma tissues in comparison with normal brain tissues (Figure 1B, *P* < 0.01). Besides, Kaplan-Meier survival curve was adopted to analyze the link between FOXN3 mRNA expression and overall survival time of glioma patients. Kaplan-Meier survival curve manifested that glioma patients with low FOXN3 expression (*n* = 32) exhibited a shorter overall survival time than those with high FOXN3 expression (*n* = 31; Figure 1C, *P* < 0.05). Collectively, these results suggest that decreased FOXN3 expression correlates with glioma patients’ poor prognosis.

### FOXN3 represses glioma cell proliferation, survival and motility

**Figure 2:**
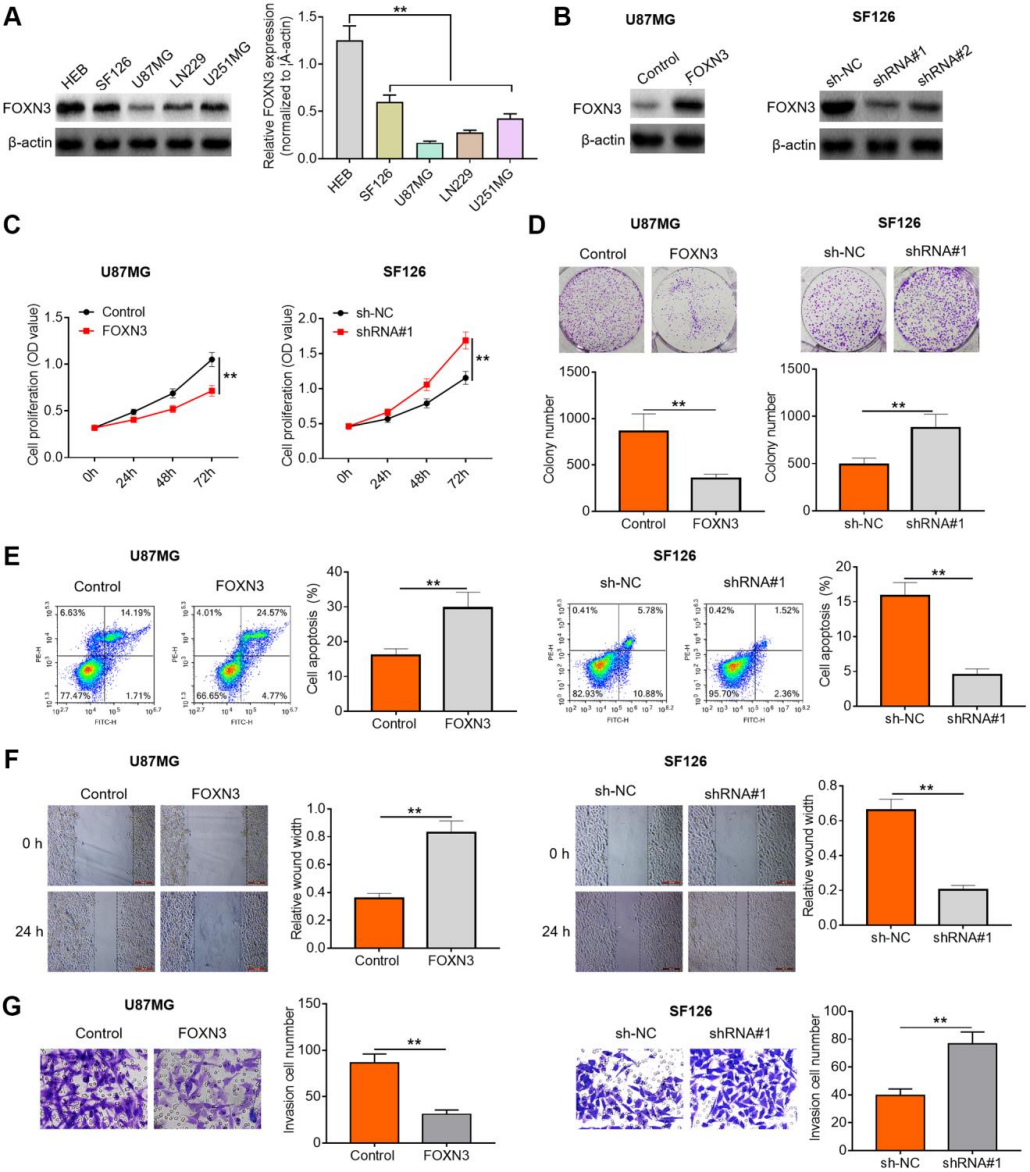
**FOXN3 suppresses glioma cell proliferation, survival and invasion.** (**A**) FOXN3 protein expression levels in normal human L02 liver cells and glioma cell lines (SF126, SK-Hep-1, Huh6 and Huh7) were determined by Western blotting. (**B**) Transfection efficiency was detected via Western blotting after transfection with FOXN3 expression vectors in U87MG cells or transfection with specific shRNAs in SF126 cells. (**C**) Cell proliferation was measured by MTT assays after transfection with FOXN3 expression vectors in U87MG cells or transfection with shRNA#1 in SF126 cells. (**D**) Colony formation ability was analyzed using colony formation assays after transfection with FOXN3 expression vectors in U87MG cells or transfection with shRNA#1 in SF126 cells. (**E**) Apoptosis was assessed via flow cytometry after transfection with FOXN3 expression vectors in U87MG cells or transfection with shRNA#1 in SF126 cells. (**F**) Migration capability was evaluated by scratch wound healing assays after transfection with FOXN3 expression vectors in U87MG cells or transfection with shRNA#1 in SF126 cells. (**G**) Invasion ability was determined via Transwell invasion assays after transfection with FOXN3 expression vectors in U87MG cells or transfection with shRNA#1 in SF126 cells. ^**^P < 0.01. FOXN3, forkhead box N3; shRNA#1, short hairpin RNA #1 specifically targeting FOXN3; shRNA#2, short hairpin RNA #2 specifically targeting FOXN3.

To further characterize the possible role of FOXN3 in human glioma, we performed subsequent cell experiments. Herein, FOXN3 protein expression patterns in glioma cell lines (SF126, U87MG, LN229 and U251MG cells) and normal human gliocytes (HEB cells) were assessed via Western blotting. As exhibited in Figure 2A, FOXN3 protein expression was significantly decreased in the glioma cell lines compared with HEB cells. Afterwards, U87MG with lowest endogenous FOXN3 expression and SF126 cells with highest endogenous FOXN3 expression were selected for the following gain- and loss-of-function researches. Subsequently, Western blotting analysis was conducted to determine the transfection efficiency after treatment with FOXN3 expression vectors or specific shRNAs (shRNA#1 and shRNA#2). As shown in Figure 2B, transfection of FOXN3 expression vectors remarkably elevated FOXN3 protein expression levels in U87MG cells in comparison with the control group; whereas transfection of shRNA#1 and shRNA#2 markedly decreased the expression levels of FOXN3 in SF126 cells in comparison with sh-NC group, especially shRNA#1. Thus, shRNA#1 was chosen for further knockdown studies. As evidenced by CCK8 assay and colony forming assay, FOXN3 over-expression significantly inhibited the proliferation of U87MG cells (*P* < 0.01), while FOXN3 knockdown sharply accelerated the proliferation of SF126 cells (Figure 2C and 2D, *P* < 0.01). Moreover, flow cytometry analysis showed that FOXN3 over-expression induced a higher proportion of apoptotic cells than the control treatment (*P* < 0.01), whereas FOXN3 ablation triggered a lower proportion of apoptotic cells than the negative control treatment (Figure 2E, *P* < 0.01). Besides, scratch assay and Transwell invasion assay showed that migration and invasion abilities of U87MG cells were significantly weakened by FOXN3 over-expression treatment, while migration and invasion abilities of SF126 cells were sharply strengthened in FOXN3 depletion group in comparison with those in sh-NC group (Figure 2F and 2G, *P* < 0.01). To sum up, these data imply that FOXN3 could represses glioma cell proliferation, survival and motility abilities.

### FOXN3 modulates the expression of malignant phenotype-associated proteins in glioma cells

**Figure 3:**
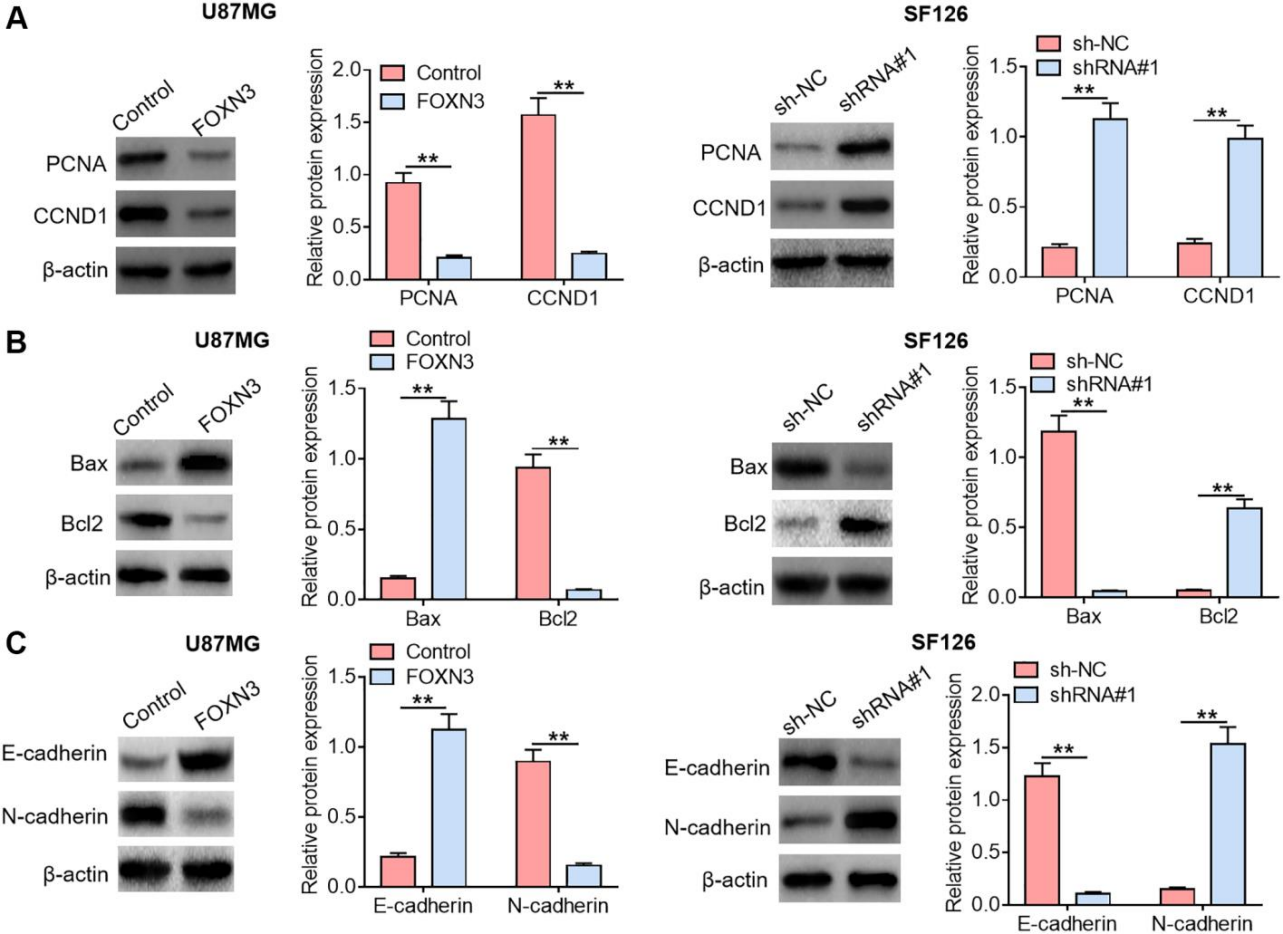
**FOXN3 modulates the expression of malignant phenotype-associated proteins in glioma cells.** (**A**) Protein expression levels of PCNA and CCND1 were examined by Western blotting after transfection with FOXN3 expression vectors in U87MG cells or transfection with shRNA#1 in SF126 cells. (**B**) Protein expression levels of cleaved caspase 3 and Bcl2 were detected via Western blotting after transfection with FOXN3 expression vectors in U87MG cells or transfection with shRNA#1 in SF126 cells. (**C**) Protein expression levels of Vimentin and MMP9 were determined using Western blotting after transfection with FOXN3 expression vectors in U87MG cells or transfection with shRNA#1 in SF126 cells. ^**^P < 0.01. FOXN3, forkhead box N3; shRNA#1, short hairpin RNA #1 specifically targeting FOXN3; Abbreviations: PCNA: proliferating cell nuclear antigen; CCND1: cyclin D1; Bcl2: B cell lymphoma; MMP9: matrix metalloproteinase 9.

It is widely accepted that some crucial functional proteins are involved in cancer occurrence and development. In order to further analyze the impacts of FOXN3 on tumor cell proliferation, apoptosis and motility, the expression patterns of malignant phenotype-related proteins in U87MGcells and SF126 cells were also evaluated. Herein, the influences of FOXN3 over-expression and knockdown on the expression levels of proliferation-related proteins (PCNA and CCND1) were analyzed by Western blotting. As displayed in Figure 3A, reduced PCNA and CCND1 protein expression levels were found in FOXN3 over-expression group in comparison with those in control group (*P* < 0.01), whereas elevated PCNA and CCND1 protein expression levels were observed in FOXN3 ablation group compared with those in sh-NC group (*P* < 0.01). Furthermore, FOXN3 over-expression elicited a noticeable elevation in cleaved caspase-3 protein expression level and a remarkable reduction in Bcl2 protein expression level in U87MG cells in comparison with control group (*P* < 0.01), while FOXN3 depletion led to increased Bcl2 protein expression and decreased cleaved caspase-3 protein expression in SF126 cells (Figure 3B, *P* < 0.01). In addition, FOXN3 over-expression inhibited the expression of MMP9 and Vimentin proteins in U87MGcells, whereas FOXN3 depletion boosted the expression of MMP9 and Vimentin proteins in SF126 cells (Figure 3C, *P* < 0.01). Taken together, these observations indicate that FOXN3 repressed the expression of malignant phenotype-promoting proteins in glioma cells.

### FOXN3 inhibits the AKT/MDM2/p53 signaling axis in glioma cells

**Figure 4:**
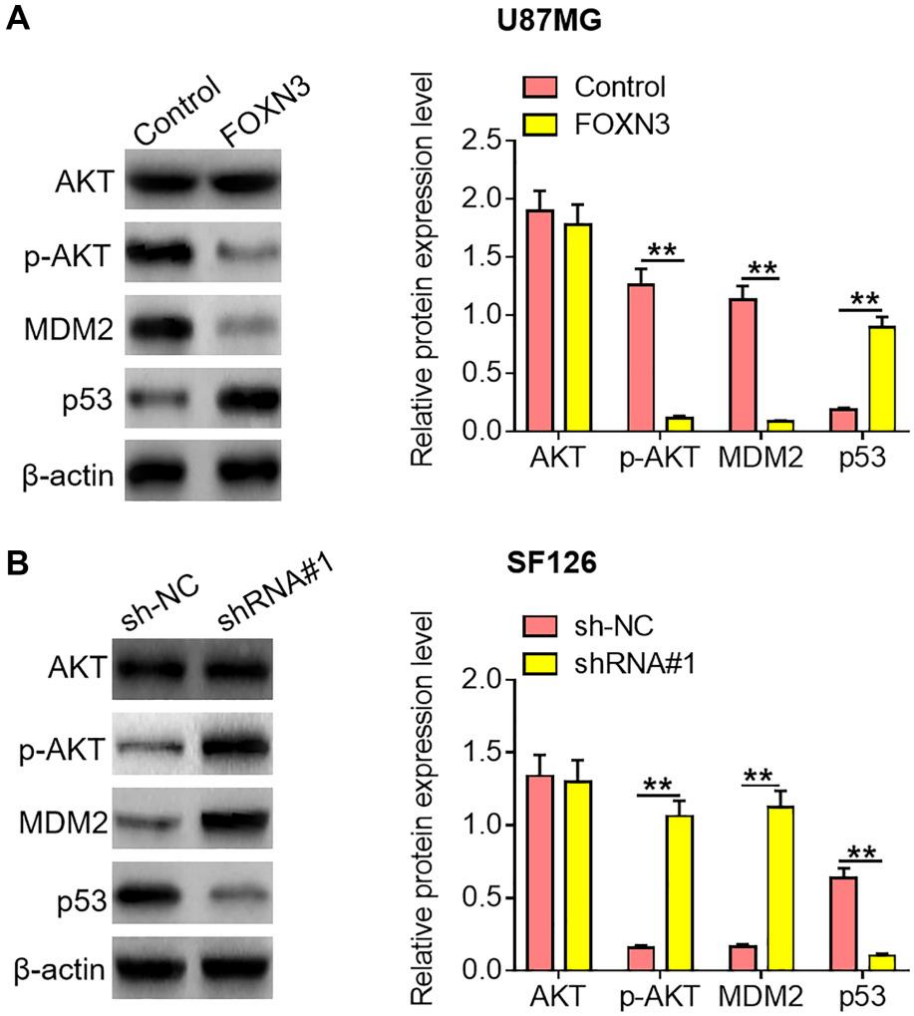
**FOXN3 inhibits the AKT/MDM2/p53 signaling pathway in glioma cells.** (**A**) Protein expression levels of AKT, p-AKT, MDM2 and p53 were examined by Western blotting after transfection with FOXN3 expression vectors in U87MG cells. (**B**) Protein expression levels of AKT, p-AKT, MDM2 and p53 were determined by Western blotting after transfection with shRNA#1 in SF126 cells. ^**^P < 0.01. FOXN3, forkhead box N3; shRNA#1, short hairpin RNA #1 specifically targeting FOXN3; Abbreviations: p-AKT: phosphorylated AKT; MDM2: murine double minute 2.

Emerging evidence has revealed that AKT/MDM2/ p53 signaling transduction would contribute to the malignant development of diverse kinds of malignant cancers. Herein, the impacts of FOXN3 over-expression and knockdown on AKT/MDM2/p53 signaling axis were assessed in glioma cells. As exhibited in Figure 4A, reduced p-AKT and MDM2 protein expression levels as well as elevated p53 protein expression level were observed in FOXN3 over-expression group in comparison with control group (*P* < 0.01). Besides, increased p-AKT and MDM2 protein expression levels as well as decreased p53 protein expression level were found in FOXN3 depletion group in comparison with sh-NC group (Figure 4B, *P* < 0.01). These results imply that FOXN3 suppresses AKT/MDM2/p53 signaling transduction in glioma cells.

### FOXN3 over-expression retards glioma cell growth *in vivo*

**Figure 5:**
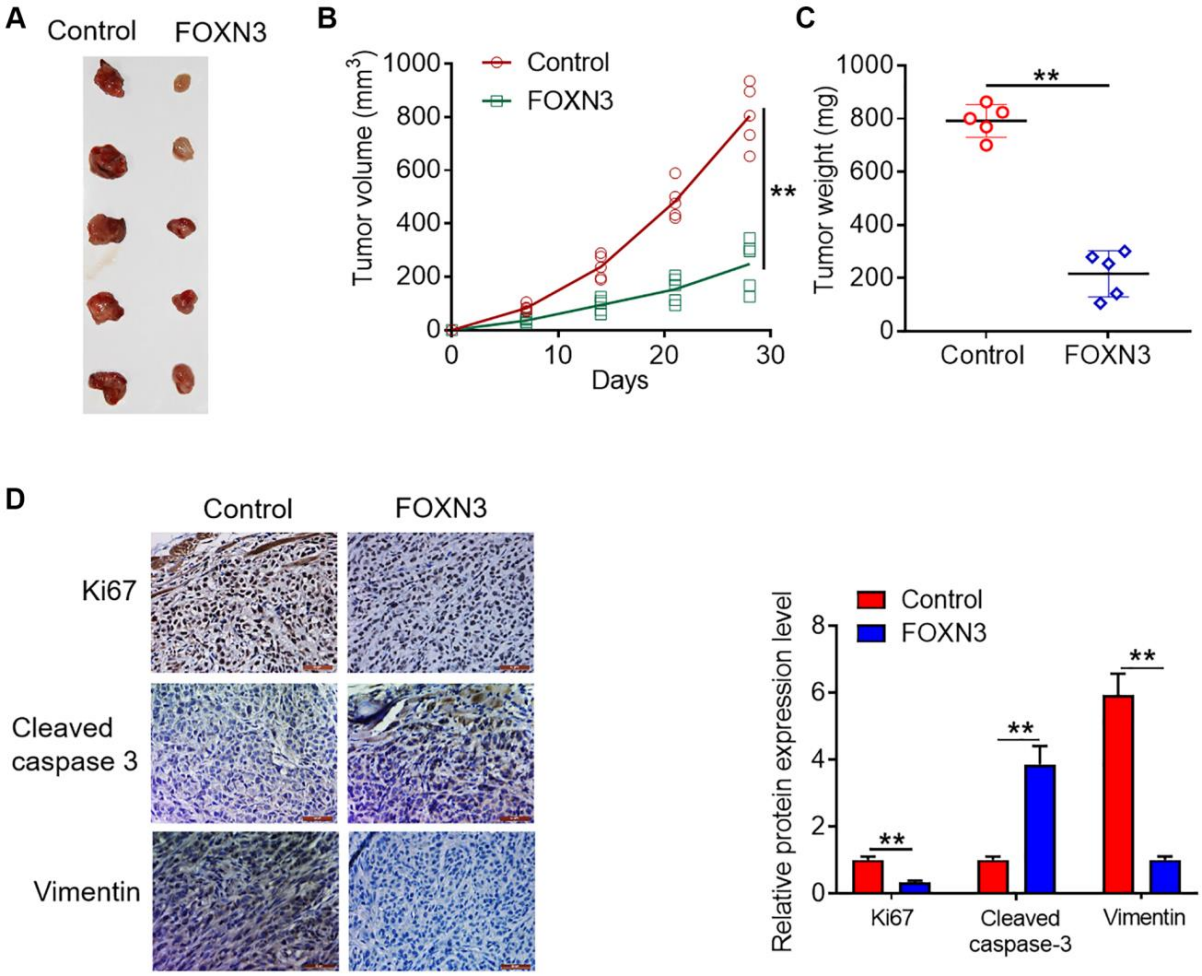
**FOXN3 over-expression retards glioma cell growth *in vivo*.** (**A**) Representative images of tumors harvested from murine neoplasm xenograft models. (**B**) Tumor growth curve was plotted based on the formula that volume (mm^3^) = [length (mm) × width^2^ (mm^2^)]/2. (**C**) Neoplasms were collected and weighed at day 28 post-transplantation. (**D**) Protein expression patterns of Ki67, cleaved caspase 3 and Vimentin in the tumors harvested from nude mice were visualized via immunohistochemical staining. ^**^P < 0.01. Abbreviation: FOXN3: forkhead box N3.

On the basis of the observations *in vitro*, the tumor transplantation models were established in mice through the subcutaneous inoculation of empty vector- or FOXN3 expression vector-treated U87MGcells into the flanks of mice. As presented in Figure 5A and 5B, the tumors in FOXN3 over-expression group grew marked slower than those in control group (*P* < 0.01). Moreover, significantly smaller neoplasms were harvested from the FOXN3 over-expression group in comparison with control group (Figure 5A and 5C, *P* < 0.01). In addition, the expression patterns of Ki67, cleaved caspase-3 and Vimentin proteins in the collected tumors from nude mice were visualized by immunohistochemical staining. As displayed in Figure 5D, less Ki67-positive and Vimentin-positive cells were found in the tumors in FOXN3 over-expression group in comparison with those in control group (*P* < 0.01). In addition, more cleaved caspase 3-positive cells were observed in FOXN3 over-expression group in comparison with control group (Figure 5D, *P* < 0.01). These data demonstrate that FOXN3 over-expression retards glioma cell growth *in vivo*, which was in consistence with the findings *in vitro*.

## DISCUSSION

It is widely accepted that glioma has aroused widespread public concerns around the world due to its high morbidity and mortality in recent decades [17–19]. It is worth noting that glioma has already imposed huge economic burdens and heath pressures on the glioma patients worldwide. Even though some significant achievements concerning the diagnosis and therapy have been gained, the overall survival of most glioma patients remains very depressing. Therefore, there is an urgent need to broaden our knowledge concerning the underlying mechanisms of glioma occurrence and progression, which would facilitate to develop promising and effective treatment alternatives. Mounting reports have manifested that the aberrant expression of tumor-related crucial genes is closely linked to the malignant progression of glioma [20–22].

In the previous studies, FOXN3 has been proved as a key player during the malignant development of varied kinds of malignant tumors. Xue et al. determined the expression pattern of FOXN3 in osteosarcoma and demonstrated that decreased FOXN3 expression repressed tumor cell invasion and migration through transcriptionally regulating SIRT6 [10]. He and his research team reported a notable decrease in FOXN3 expression in adult acute myeloid leukemia and their data revealed that FOXN3 exerted tumor-suppressing roles by inhibiting cell proliferation and promoting apoptosis [11]. Zhao et al. reported that decreased FOXN3 expression was observed in papillary thyroid cancer and discovered that FOXN3 inhibited tumor cell growth and invasion via the inactivation of Wnt/ β-catenin pathway [13]. Moreover, researches by Chen et al. [14] and Sun et al. [15] manifested that FOXN3 repressed tumor cell proliferation and epithelial-to-mesenchymal transition as well as metastasis in melanoma and tongue squamous cell carcinoma. Nonetheless, the biological role of FOXN3 remains poorly understood.

In the current study, FOXN3 expression patterns in glioma tissues were characterized via qPCR analysis and Western blotting. Herein, it was found that FOXN3 was notably under-expressed in the tumorous tissues and that reduced FOXN3 expression correlated with short overall survival time of patients with glioma. Moreover, gain of function and loss of function studies showed that up-regulation of FOXN3 notably inhibited glioma cell proliferation, survival and motility, while down-regulation of FOXN3 dramatically facilitated glioma cell proliferation, survival and invasion. In addition, our findings demonstrated that FOXN3 over-expression triggered an obvious reduction in PCNA, CCND1, MMP9 and Vimentin expression levels in glioma cells, whereas FOXN3 ablation elicited a remarkable elevation in PCNA, CCND1, MMP9 and Vimentin expression levels. It is well documented that decreased PCNA and CCND1 expression is associated with retarded proliferation of tumor cells [23–25] and that reduced MMP9 and Vimentin expression is closely related to slowed invasion of cancer cells [26, 27].

With the purpose of illuminating the potential molecular mechanisms underlying the biological functions of FOXN3 in glioma, further mechanistic studies were performed. Past researches have confirmed that the abnormal activation of the AKT/MDM2/p53 signaling axis is implicated in several types of human malignant neoplasms [28–30]. In the present research, we assessed the effects of FOXN3 on AKT/MDM2/p53 signaling axis in glioma cells. Herein, it was observed that FOXN3 over-expression repressed AKT/MDM2/p53 signaling transduction in glioma cells, whereas FOXN3 ablation facilitated the activation of AKT/MDM2/p53 signaling axis. It is widely acknowledged that AKT phosphorylation could facilitate the stabilization and nuclear translocation of the transcription factor MDM2, thereby triggering subsequent ubiquitination and degradation of the tumor suppressor factor p53 [31–33]. It is worthwhile to note that the inactivation of AKT/MDM2/p53 is regarded as a promising treatment option against human malignancies [34, 35]. In addition, *in vivo* xenograft assays demonstrated that FOXN3 over-expression could restrain tumor growth in the nude mice models.

To sum up, our findings for the first time demonstrated that FOXN3 was markedly under-expressed in human glioma and that reduced FOXN3 expression was linked to short overall survival time of patients with glioma. Moreover, functional and mechanistic researches preliminarily revealed that FOXN3 suppressed proliferation, survival and invasion of glioma cells through inactivating the AKT/MDM2/p53 axis. Hence, the present study may offer some valuable clues for clarifying the possible molecular mechanisms underlying glioma development and progression.
